# Giant Complex Odontoma of the Posterior Mandible: A Case Report

**DOI:** 10.7759/cureus.70402

**Published:** 2024-09-28

**Authors:** Arun S Dodamani, Manish Sharma, Seema Gupta, Charu Singhal

**Affiliations:** 1 Department of Public Health Dentistry, Jawahar Medical Foundation's Annasaheb Chudaman Patil Memorial Dental College, Dhule, IND; 2 Department of Oral Pathology, Jawahar Medical Foundation's Annasaheb Chudaman Patil Memorial Dental College, Dhule, IND; 3 Department of Orthodontics, Kothiwal Dental College and Research Centre, Moradabad, IND; 4 Department of Oral Pathology, Kothiwal Dental College and Research Centre, Moradabad, IND

**Keywords:** benign, giant, hamartoma, odontogenic tumor, odontoma

## Abstract

Complex odontoma is a benign odontogenic tumor composed of disorganized dental tissues, including enamel, dentin, cementum, and pulp. Unlike compound odontomas, which exhibit tooth-like structures, complex odontomas form a mass without anatomical organization. These tumors frequently present without symptoms and are typically identified inadvertently during standard dental imaging procedures. They originate from the dental lamina and develop due to tooth-development disturbances, potentially influenced by genetic, developmental, and environmental factors. They are most commonly found in the posterior mandible. Although odontomas are the most frequent odontogenic tumors, complex odontomas remain relatively rare. Clinically, they may cause delayed tooth eruption, swelling, or displacement of adjacent teeth. Radiographically, complex odontomas appear as radiopaque masses with a surrounding radiolucent halo. Histopathologically, they consist of a disorganized mix of dental tissues. Differential diagnoses include other odontogenic tumors such as ameloblastic fibro-odontoma and calcifying epithelial odontogenic tumors. This case report aims to present a rare instance of giant complex odontoma in a 17-year-old male patient, detailing its clinical, radiographic, and histopathologic features. Emphasizing the importance of early detection, this report highlights the role of proper diagnosis and timely intervention to ensure optimal treatment outcomes.

## Introduction

Complex odontoma is a benign odontogenic tumor composed of disorganized dental tissues, including enamel, dentin, cementum, and pulp. Unlike compound odontomas, which display tooth-like structures, complex odontomas form an irregular mass without recognizable organization [1,2]. These pathological formations are characterized by non-aggressive behavior and a gradual proliferation, frequently identified inadvertently during standard dental assessments owing to their lack of symptoms [3]. Complex odontomas originate from odontogenic tissues, specifically from remnants of the dental lamina involved in tooth development [1,4]. The precise mechanism behind their development remains unclear. However, it is hypothesized that disruptions during tooth formation result in the improper differentiation and arrangement of dental tissues, leading to the formation of these tumors [5].

In terms of prevalence, odontomas are the most common type of odontogenic tumors, accounting for approximately 22% of all cases, with complex odontomas being less frequent than compound odontomas [6]. The etiology of complex odontomas is thought to be multifactorial, involving genetic, developmental, and environmental factors. Local trauma, infections, and inflammatory processes during tooth development are common contributing factors [5,6]. Additionally, genetic conditions such as Gardner syndrome and basal cell nevus syndrome have been associated with an increased likelihood of odontoma formation, indicating a potential hereditary predisposition [7,8]. Odontomas are common in permanent dentitions and rarely found in primary dentitions [9].

Complex odontomas are systematically classified in conjunction with compound odontomas; however, they exhibit distinct histopathologic and radiographic characteristics. The most contemporary classification established by the World Health Organization (WHO) categorizes odontomas as hamartomatous lesions rather than authentic neoplasms, thereby underscoring their classification as developmental anomalies instead of tumors. The WHO's 2017 classification of odontogenic tumors further delineates compound and complex odontomas predicated on their structural organization and radiographic presentation [10,11]. The absence of structured tooth morphology is a key distinguishing feature of complex odontoma [8,12].

Differential diagnosis for complex odontomas includes other odontogenic tumors such as ameloblastic fibro-odontoma, calcifying epithelial odontogenic tumor, and ossifying fibroma [1,6,12,13]. Accurate diagnosis requires a combination of clinical, radiographic, and histopathologic evaluation. This case report aims to present a rare instance of a complex odontoma, highlighting its clinical and radiographic features and discussing the diagnostic and therapeutic approaches. This report emphasizes the importance of early detection and treatment of odontomas to prevent complications such as delayed tooth eruption and dental crowding, contributing to better clinical outcomes.

## Case presentation

**Figure 1:**
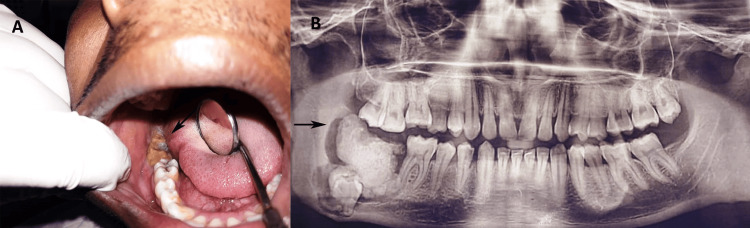
Clinical and radiological features (A) bony, hard yellowish-brown mass distal to the first molar on the right side of the mandible. (B) The OPG shows a radiopaque mass surrounded by a radiolucent rim and is associated with an impacted second molar at the lower border of the mandible. OPG: orthopantomogram

A 17-year-old male subject was referred to the Oral and Maxillofacial Pathology Department at Jawahar Medical Foundation ACPM Dental College in July 2023, complaining of enduring pressure and discomfort localized in the right posterior region of the mandible. The subject's medical history was insignificant, devoid of any systemic pathologies, and laboratory evaluations did not reveal any anomalies. There was an absence of any reported discharge of blood or pus from the affected area. An extraoral examination yielded no indications of inflammation or cervical lymphadenopathy. Conversely, an intraoral examination discerned a well-circumscribed hard swelling located in the mandibular vestibule on the right side, characterized by a yellowish-brown lesion manifesting as an irregular erupting calcified mass, alongside the clinical absence of the second and third molar teeth (Figure 1A).

An orthopantomogram (OPG) (Pax-I, Vatech, India) demonstrated an amorphous, radiopaque lesion measuring approximately 3.8 × 4.2 cm, surrounded by a radiolucent margin, and causing obliteration of the inferior margin of the mandible. The lesion was correlated with marked osseous expansion in the region of the inferior alveolar nerve (IAN) on the right side of the posterior mandible. Furthermore, the mass impeded the eruption trajectory of the impacted second molar, which displayed a mesioangular position (Figure 1B).

A preliminary diagnosis of complex odontoma was proposed for this case based on the clinical and radiographic characteristics. The lesion was surgically removed under general anesthesia. Access to the mass was obtained through an intraoral approach, and the odontoma was elevated using a periosteal elevator. The capsule surrounding the lesion was carefully curetted. The odontoma was observed as an in-toto entity. The excised specimen weighed 90.5 g and was characterized by a rough, stony-hard consistency.

**Figure 2:**
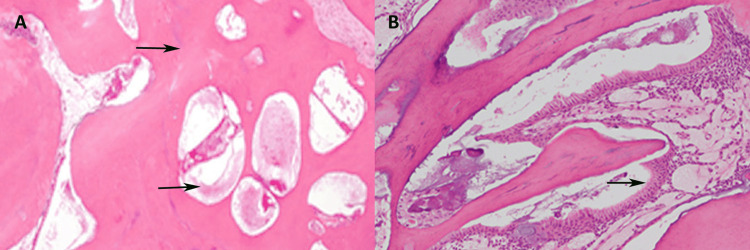
Histopathological features: (A) a complex odontoma with sheets of calcified tissue enamel and dentin, along with areas of mature pulp tissue; (B) an area showing ectomesenchymal tissue with ameloblastic lining at the periphery.

The specimen was subjected to histopathological examination after the decalcification using the hematoxylin and eosin (H&E) staining technique. Observations under light microscopy (Labomed, India) at magnifications of 40× (Figure 2A) and 100× (Figure 2B) demonstrated a disorganized configuration of dental tissues, encompassing tubular dentin, cementoid regions, enamel matrix, and pulp-like connective tissue, all embedded within a collagen fiber matrix containing vascular structures. The enamel component was compromised during the decalcification procedure. These observations corroborated the preliminary diagnosis.

The postoperative course was smooth. The patient completed a six-month follow-up period without any signs of tumor recurrence. No complications or infections were observed during the follow-up visits.

## Discussion

Complex odontomas are predominantly found in the posterior regions of the mandible, whereas compound odontomas more commonly occur in the anterior maxilla. The estimated incidence of complex odontomas ranges from 5% to 37% of all odontogenic tumors [1,4,6]. While they can develop at any age, they are most frequently diagnosed in young adults, often before the age of 30 [2,4]. While odontomas may manifest in various locations within the jaws, compound odontomas are predominantly observed in the canine and incisor areas of the maxillary arch [2]. In contrast, complex odontomas are more frequently located in the mandibular molar region [12]. It is typically small in size and asymptomatic. However, in the present case report, it was large in size, measuring 3.8 × 4.2 cm. Due to its size exceeding 3 cm, it was diagnosed as a giant odontoma, according to a previous study [14]. Park et al. reported a similar case of a complex giant odontoma in a 28-year-old female patient affecting the right side of the posterior mandible, measuring 30 × 25 × 20 mm [14]. Typically, giant odontomas do not exceed 3 cm in size due to their restricted development capacity; however, in our case, the odontoma grew 4.2 cm.

A substantial odontoma, reaching dimensions of up to 6 cm, has been documented, with the heaviest odontoma recorded at 0.3 kg [15]. Research conducted by Miki et al. revealed that merely 4.3% of odontomas exceeded 3 cm in size [16]. The tooth may be clinically unerupted or absent. This phenomenon was similarly observed in the current case, which is characterized as an impacted right mandibular second molar.

Odontomas can be classified as central (intraosseous) or peripheral (extraosseous) in nature [1]. The current case under discussion pertains to an intraosseous odontoma, which manifests entirely within the jaw structures, resulting in the expansion of the surrounding bone. Moreover, odontomas have the potential to induce bone resorption, particularly when the lesion exhibits an increase in size. While an odontoma is classified as a hamartoma, the suggested therapeutic intervention entails conservative surgical excision followed by histopathological analysis of the lesion to distinguish it from other pathological entities. Notably, there exists a limited number of documented instances of recurrence subsequent to excision, and the overall prognosis remains favorable [15]. In the present case report, after the surgical removal of the odontoma, the recurrence was not noticed at six months follow-up.

The differential diagnosis for complex odontoma encompasses a diverse array of lesions characterized by radiopaque masses encircled by radiolucent margins [3]. Radiographically, complex odontomas may mimic conditions such as ossifying fibroma, osteoblastoma, and osteoma [6]. Radiologically, complex odontomas appear as well-defined radiopaque masses with irregular shapes, often surrounded by a radiolucent halo, without identifiable tooth-like structures. They are typically located in the posterior jaws and can cause tooth displacement. Histopathologically, complex odontomas consist of disorganized dental tissues, including enamel, dentin, cementum, and pulp, arranged in a haphazard manner and encapsulated by fibrous connective tissue. This distinguishes them from other odontogenic tumors and lesions, such as ameloblastic fibro-odontomas or cementoblastomas, which have different structural organization and cellular features. Generally, the prognosis for odontomas is exceedingly favorable, exhibiting a very rare propensity for recurrence [17].

Despite the fact that panoramic X-rays exhibit a multitude of recognized limitations as being two-dimensional, it is imperative to acknowledge that the routine implementation of dental panoramic imaging can play a critical role in the incidental detection of these often-asymptomatic lesions prior to their progression into more significant, expansive forms that may lead to noticeable jaw enlargement and facial asymmetry. The identification of such lesions at an earlier stage has the potential not only to facilitate timely surgical intervention for the removal of these odontomas but also to minimize the associated morbidity and complications that can arise, including, as was unfortunately the case here, the loss of permanent teeth. In a fortunate turn of events, it is noteworthy to mention that the patient in question did not experience any neurological complications following the surgical procedure, despite the considerable size of the lesion and its consequential inferior displacement of the mandibular canal that could have led to such adverse outcomes. This underscores the significance of early detection through panoramic imaging, which can serve as a vital tool in mitigating the risks associated with the surgical management of odontogenic tumors.

The limitation of our case report is the absence of a long-term follow-up and the use of a three-dimensional (3D) cone-beam computed tomographic (CBCT) examination of the lesion. CBCT analysis in a large complex odontoma case of the posterior mandible offers precise 3D visualization, accurate localization, and detailed assessment of the lesion's impact on adjacent teeth and bone. It aids in safer surgical planning, reduces radiation exposure, and enhances post-surgical monitoring for optimal outcomes.

## Conclusions

This case report elucidates a rare instance of a giant mandibular complex odontoma, thereby enhancing comprehension of the clinical characteristics associated with such extensive lesions in the mandible. Giant complex odontomas can manifest at any stage of life without a discernible gender bias, and they may be linked to discomfort or sensitivity, frequently resulting in bone proliferation and facial asymmetry. Typically, they exhibit a favorable prognosis, and meticulous surgical excision is regarded as a reliable therapeutic approach.
